# Analysis of volatile organic compounds in biological samples of colorectal cancer patients using electronic nose-based machine learning techniques

**DOI:** 10.1038/s41598-025-27529-1

**Published:** 2025-12-25

**Authors:** Nada E. Ahmed, Mohamed S. Mshaly, Khaled M. Madbouly, Marwa A. Mohamed, Ebtsam A. Abdel-Wahab, Ehab I. Mohamed

**Affiliations:** 1 Medical Biophysics Department, Medical Research Institute, Alexandria University, 165 El-Horreya Avenue, Alexandria, 5433005 Egypt; 2 Physics Department, Faculty of Science, Alexandria University, Alexandria, Egypt; 3 Sugery Department, Faculty of Medicine, Alexandria University, Alexandria, Egypt; 4 Chemical Pathology Department, Medical Research Institute, Alexandria University, Alexandria, Egypt; 5 Biomedical Equipment Technology Department, Faculty of Applied Health Sciences Technology, October 6 University, Giza, Egypt

**Keywords:** Colorectal cancer (CRC), Cancer screening, Volatile organic compounds (VOCs), Headspace analysis, Electronic nose (eNose), Machine learning (ML), Biomarkers, Cancer, Computational biology and bioinformatics, Gastroenterology, Oncology

## Abstract

Colorectal cancer (CRC) is a significant global health burden characterized by prolonged asymptomatic progression and high mortality. CRC curability improves with early-stage detection, and removing precancerous adenomas allows for prevention, emphasizing the significance of screening. This prospective study, conducted between 2024 and 2025 with 100 randomly recruited participants, investigates eNose-based analysis of volatile organic compounds (VOCs) in biological matrices for CRC diagnosis using both unsupervised and supervised machine learning (ML) techniques. After detailed medical examinations, laboratory tests, and colonoscopy, 50 patients with confirmed stage III CRC and 50 healthy controls agreed to have their blood, urine, and stool samples analyzed by the eNose technique. Principal component analysis (PCA), logistic regression (LR), k-nearest neighbor (KNN), support vector machine (SVM), and gradient boosting (GB) were used to analyze eNose VOC patterns in all biological matrices. Clinical and hematological alterations in CRC patients were consistent with systemic malignancy, including reduced weight, mild anemia, leukopenia, thrombocytopenia, and hypoalbuminemia, all of which are established indicators of disease severity and prognostic markers. Elevated VOC responses in CRC patients across all matrices, with blood and stool proving most informative due to favorable signal-to-noise ratios. Ensemble- and proximity-based models GB and KNN were found to be superior to LR classifiers, with GB exhibiting balanced and adaptable performance across different biological matrices. Limiting the study to stage III CRC patients improved VOC signal clarity but limited early-stage generalizability, a constraint effectively mitigated by Gaussian augmentation, which enriched data variability and boosted model performance for screening applications. Thus, eNose-based ML systems provide a globally accessible, innovative, non-invasive, and affordable solution for CRC detection, combining high sensitivity and specificity to support widespread early diagnosis.

## Introduction

Colorectal cancer (CRC) begins as benign adenomatous polyps that progress through the adenoma–carcinoma sequence, typically over 10–15 years, by acquiring gradual genetic and epigenetic changes from normal mucosa to early and advanced adenomas, carcinoma in situ, and ultimately invasive carcinoma^1^. This transformation is influenced by hereditary syndromes, environmental exposures, and lifestyle factors. While CRC is preventable and treatable when detected early, it is the third most common cancer, accounting for 10% of all cancer cases and the second highest cause of cancer deaths globally^2^. In 2020, CRC reported 1.9 million new cases and 930,000 deaths, figures that are estimated to increase to 3.2 million new cases and 1.6 million deaths by 2040^3^. Moreover, the early-onset CRC (< 50 years) is rising, especially in urban populations, with higher incidence in high-income countries like Australia, New Zealand, and parts of Europe^4^. In 2022, approximately 5,940 new cases and 3,096 deaths were caused by CRC in Egypt, making it seventh in incidence and ninth in mortality overall, with an age-standardized incidence rate of 9.8 per 100,000 individuals^5,6^. Notably, the early-onset CRC is exceptionally high in Upper Egypt, as are late-stage and rectal malignancies, due to the lack of screening and delayed diagnosis^7,8^.

Early detection of CRC can lower mortality rates, making it crucial to develop accurate, reliable, and effective screening tools^9^. Common non-invasive screening methods like the fecal occult blood test (FOBT) have low sensitivity and specificity for early-stage CRC^10^ while established serum tumor biomarkers like carcinoembryonic antigen (CEA) and carbohydrate antigen 19−9 (CA 19−9) lack adequate sensitivity and specificity for definitive CRC diagnosis due to their presence in other malignancies^11–13^. While colonoscopy and flexible sigmoidoscopy, which must be done in a clinic, are effective and frequently recommended screening methods in developed countries, they are invasive, expensive, resource-intensive, uncomfortable, and can cause gastrointestinal complications to patients^14,15^. It is, thus, critical to have an accessible, non-invasive, affordable, convenient, highly sensitive, and accurate early CRC screening method^16^.

Endogenous volatile organic compounds (VOCs), altered by internal metabolism and external stimuli, have recently garnered interest as non-invasive biomarkers for cancer detection and monitoring^17^. Moreover, certain VOCs may contribute to carcinogenesis by promoting oxidative stress, inflammation, and dysregulated cellular processes including apoptosis and proliferation, ultimately resulting in tumorigenesis and metastasis^18^. Thus, VOCs transported via blood circulation and excreted through urine, stool, skin, and exhaled breath, reflect the patient’s metabolic and physiological state, providing clinically relevant insights into cancer development and progression^19,20^. Among these, alcohols, aldehydes, ketones, and short-chain fatty acids; represent CRC-specific biochemical alterations and constitute a non-invasive biomarker profile^21^. Notably, a panel of 15 CRC-specific VOCs were found in patients’ exhaled breath compared to healthy controls using thermal-desorption gas chromatography-mass spectrometry (TDGC-MS) and probabilistic neural network analysis with 86% sensitivity, 83% specificity, and 76% overall accuracy^22^. However, pulmonary conditions, tobacco exposure, and dietary variability may confound breath-based VOC diagnostics, therefore; profiling VOCs in blood, urine, and stool is expected to yield a more stable and accurate representation of systemic metabolic changes^16,23^.

Electronic nose (eNose) technologies are revolutionizing medical diagnostics by replicating human smell through chemically non-specific sensor arrays and advanced pattern recognition algorithms. Since the mid-1980s, eNoses have been employed in biomedical fields to identify microbial infections and pathologies, represented by sensor electrical resistance and relative changes in their conductance^24,25^. Unlike conventional GC-MS, eNose sensors can detect and recognize VOCs found in the headspace over biological samples, producing disease-specific VOC signatures for rapid and non-invasive screening^26,27^. Both supervised and unsupervised machine learning (ML) algorithms were then used to analyze sensor patterns, enabling the creation of predictive models for disease identification, real-time, and automated decision support in clinical settings^28–30^.

While their integration into medical practice remains in its preliminary stages, a recent investigation involving individuals with CRC and lung cancer suggests eNoses could contribute to early cancer diagnosis and screening^31^. In oncology, eNose systems using machine learning techniques have shown high diagnostic accuracy to classify blood-based VOC profiles for lung, breast, liver, brain, and hematologic cancers with remarkable sensitivity, specificity, and diagnostic accuracy^28^. Mohamed et al.,^32^ also showed earlier that eNose analysis of breath, blood, and urine could reliably distinguish lung cancer patients from healthy controls using principal components and artificial neural networks, achieving classification success rates up to 100%.

This study not only examines the efficacy of principal component analysis (PCA) and linear discriminant analysis (LDA) in CRC diagnosis using eNose technology but also four separate ML models: logistic regression (LR), k-nearest neighbor (KNN), support vector machine (SVM), and gradient boosting (GB).

## Patients and methods

### Study participants

One hundred adult males and females aged 20 to 65 years volunteered for this prospective study conducted between 2024 and 2025. They were chosen randomly from among patients referred to Alexandria University Main Hospital in Alexandria, Egypt, for diagnosis and/or treatment. The study’s objectives, procedures, and requirements were thoroughly communicated to all participants prior to recruitment and sample collection. All participants voluntarily agreed to participate in the study by signing written informed consent and providing blood, urine, and stool samples. Ethics Committees at both the Medical Research Institute and the Faculty of Medicine at Alexandria University in Alexandria, Egypt, approved the study protocol with Approval No. 00012098. All procedures conformed with the Declaration of Helsinki, with strict adherence to ethical and privacy regulations ensuring the confidentiality and security of all participants’ personal data throughout data handling.

### Medical examination

All participants underwent history taking, thorough medical examination, and standard laboratory testing to ascertain their health status. They also had colonoscopies, and if polyps were detected, a biopsy was taken for pathological evaluation to confirm CRC, the gold standard for diagnosis in this study^22^. Patients whose CRC was confirmed (*n* = 50) had their primary tumors’ pathological stages determined using the Cancer Staging Manual^23^. Individuals who were either verified to be CRC-free or had benign polyps via biopsy were used as controls (*n* = 50). An Excel datasheet was used to randomly assign individuals to either the ‘Colorectal Cancer’ or ‘Control’ groups by a professional colorectal endoscopist (KMM) from the Surgery Department, Faculty of Medicine, Alexandria University, Alexandria, Egypt.

### Inclusion and exclusion criteria

The study comprised controls and patients in the age range of 20–65 years who were scheduled to have their first CRC surgery. Exclusion criteria for participation in the study included a patient’s history of inflammatory bowel disease, chronic kidney disease, decompensated diabetes, or history of other malignancies; patients’ inability to communicate or provide biological samples; patients’ poor daily functioning; patients’ life expectancy being less than six months; and patients with persistently unresolved diagnoses.

## Methods

### Biological samples collection

The blood samples were taken from the participants’ upper limb peripheral veins in duplicate sterile 2 ml vacutainer tubes on EDTA (GD, Biomedica Alex Co., Alexandria, Egypt) and assigned a random number. Early morning urine and stool samples were also collected from each participant in sterile plastic cups using the same number. First tube blood samples were routinely tested for hemoglobin, red blood cell (RBC), white blood cell (WBC), and platelet counts using an Automatic Blood Cell Counter Plus CRP (HORIBA, Japan).

### eNose measurements

**Fig. 1:**
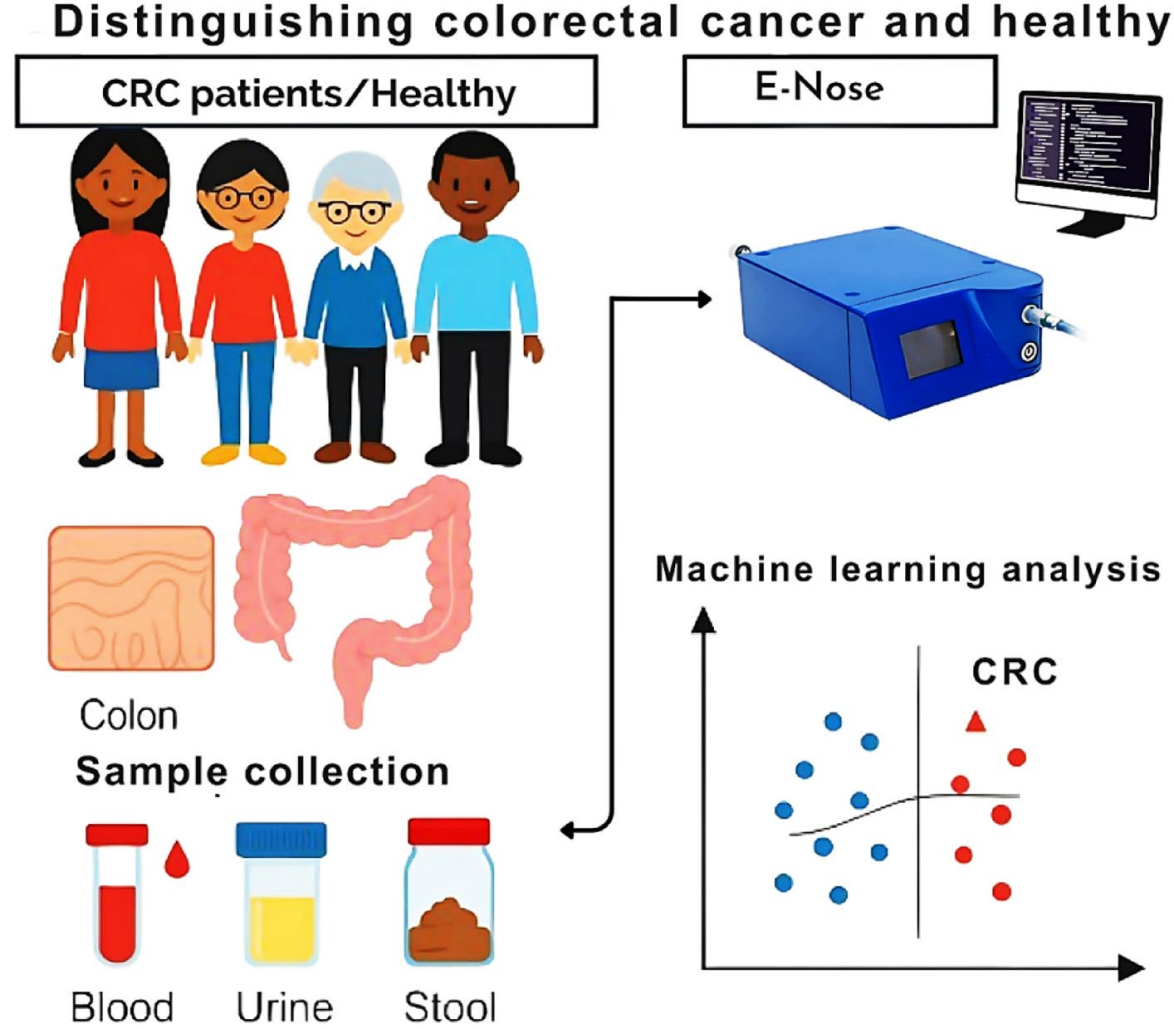
Diagram illustrating blood, urine, and stool sample collection from colorectal cancer (CRC) patients and control participants, the PEN3 eNose system, and user-friendly interface for controlling parameters and machine learning analysis.

Blood, urine, and stool samples were transported in ice boxes to the Medical Biophysics Department, Medical Research Institute, Alexandria University, and were analyzed by a single user (EIM) blinded to participants’ names or diagnoses using a portable eNose (PEN3, Airsense Analytics GmbH, Schwerin, Germany) (Fig. 1). VOCs in the headspace over samples were detected using a 10-fold array of chemically nonspecific metal-oxide semiconductor (MOS) sensors, their names and sensitivity ranges are summarized in Table 1^27,33^. A user-friendly software (WinMuster Ver. 1.6.2.24, Airsense Analytics GmbH, Schwerin, Germany) was used to manage the eNose, capture data, make calibrations, apply odor classification algorithms, and visualize results using graphs and charts^27,28,33–36^.

**Table 1:**
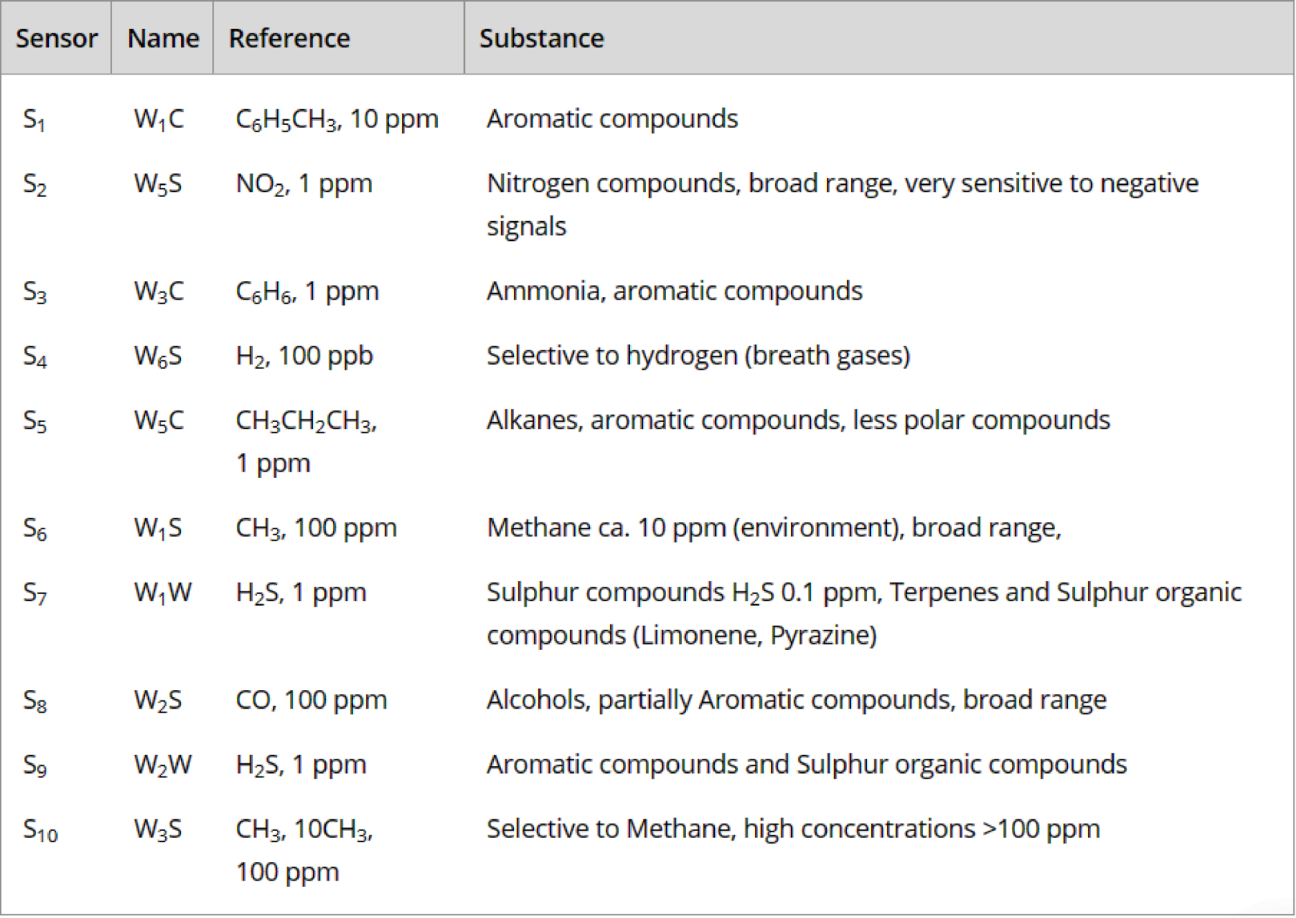
Portable Electronic Nose (PEN3, Airsense Analytics, GmbH, Schwerin, Germany) sensor brands and reference sensitivity characteristics as supplied by the manufacturer^27^.

Fresh, or refrigerated overnight at 4 °C, samples were left to equilibrate at room temperature (25 ± 1 °C) for 30 min. to ensure VOCs accumulation in the headspace, without compromising the quality of measurements^34^. Before each sample measurement, the eNose sensors underwent a 60 s cleaning phase with charcoal-filtered dry room air, followed by a 10 s zeroing period to recalibrate signals to their baseline (G/G_o_ = 1), guided by WinMuster software. For sample introduction, each sealed sample container was connected to the eNose inlet via a 3 mm internal diameter Teflon tube using a 20 G long lure-lock needle, with a shorter needle simultaneously inserted into the seal to facilitate room air entry. Headspace VOCs were then transported from each sample to the eNose sensor array by charcoal-filtered dry room air at a flow rate of 400 ml/min. During the measurement process, solenoid valves alternately directed either room air or headspace VOCs to the sensors. This automated process continuously recorded changes in sensor electrical resistance (R) and relative conductance (G/G_o_) for 60 s, saving data to a unique file for each measurement. All samples were measured in duplicates, and the resulting data files were saved in the standard (.nos) format for subsequent analysis^27,28,33–36^.

### Machine learning analysis

**Fig. 2:**
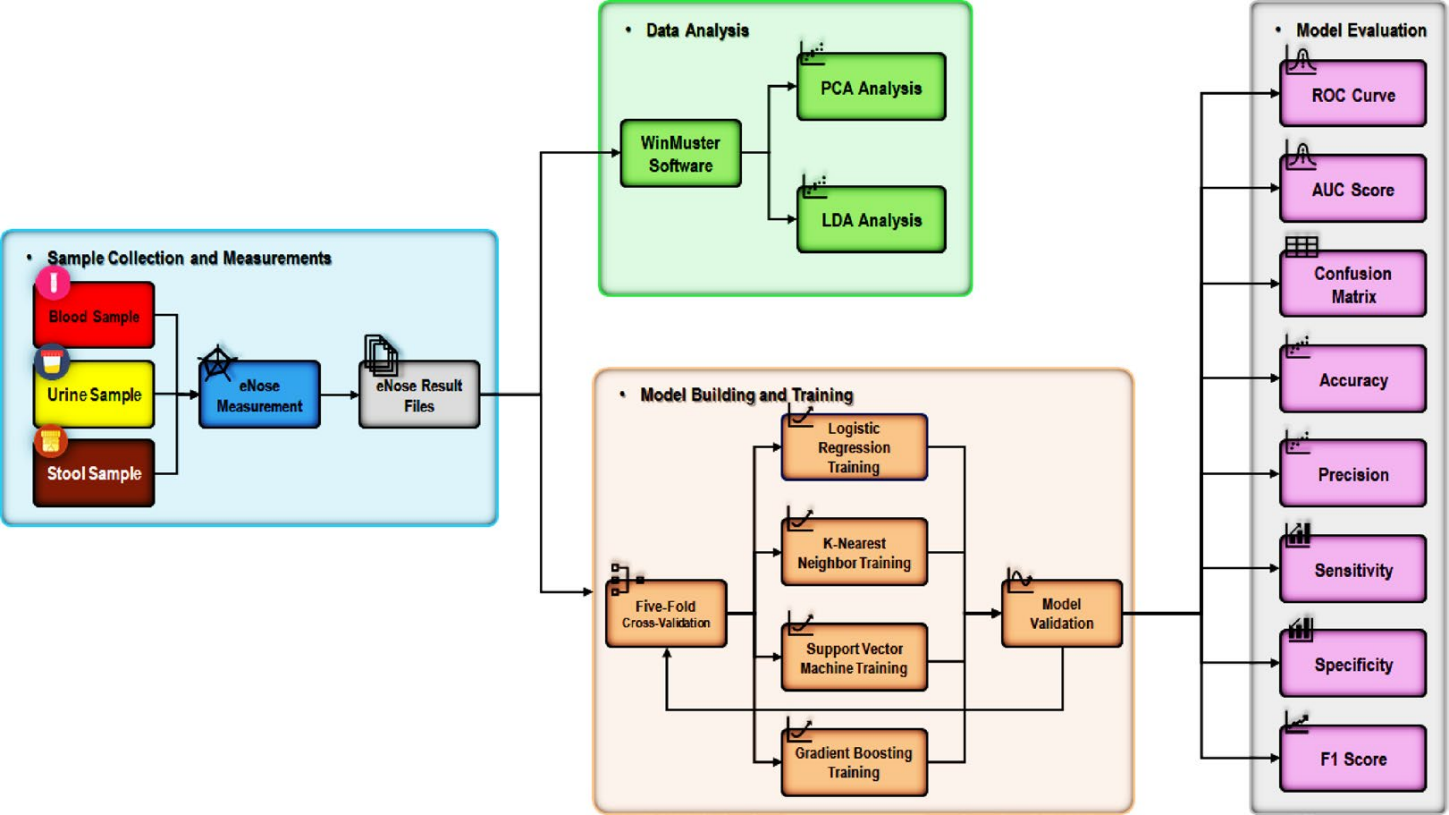
Flow diagram of the study illustrating blood, urine, and stool eNose measurements, and result files analysis using built-in algorithms: principal component analysis (PCA) and linear discriminant analysis (LDA), as well as machine learning models: logistic regression (LR), k-nearest neighbor (KNN), support vector machine (SVM), and gradient boosting (GB).

ML has significantly influenced both unsupervised and supervised automatic algorithms, enabling automated data learning, performance optimization, and informed decision-making^37,38^. ML has been applied successfully to analyze eNose sensor patterns, creating predictive models for disease identification and real-time clinical decision support^28–30,39^. Finding patterns in datasets is the first step in building predictive models using these techniques; the more data used to train the models, the more accurate the predictions will be^40^. Among various ML algorithms, PCA and LDA are commonly employed for dimensionality reduction and pattern recognition in complex datasets. PCA, an unsupervised technique, reduces data dimensionality by identifying principal components that capture the greatest variance, thereby revealing latent structures without performing classification. In contrast, LDA is a supervised method that seeks optimal linear combinations of features to maximize class separability. Both techniques are instrumental in preprocessing and visualizing high-dimensional medical data^41^. Figure 2 presents the study’s workflow, including eNose measurements from blood, urine, and stool samples, and the subsequent analysis using built-in PCA and LDA algorithms, alongside supervised ML models—LR, KNN, SVM, and GB—with their respective evaluation procedure.

eNose sensor response data files were consecutively processed using WinMuster software (Version 1.6.2.2, Airsense Analytics GmbH, Schwerin, Germany), following previously established procedures^27,28,33–36,39^. Stable sensor signals at the 50 s plateau were extracted from the 10-dimensional dataset and transformed via PCA and LDA for visualization in an *x*–*y* orthogonal coordinate system. PCA projections highlighted variance-driven clustering, while LDA plots delineated CRC and control groups based on supervised feature separation^33^.

LR, KNN, SVM, and GB—ordered by their typical runtime behavior, scalability, and resource demands—are among the most widely utilized ML algorithms in biomedical research due to their robust predictive capabilities and adaptability across diverse datasets. LR offers interpretable probabilistic outputs, making it particularly valuable for binary classification tasks such as disease risk prediction^42^. KNN provides a non-parametric approach ideal for patient similarity analysis and early disease detection, though its prediction phase can be computationally intensive due to distance calculations. SVM is renowned for its effectiveness in high-dimensional spaces, often applied in cancer classification and radiomics, though its scalability may be limited in large datasets. GB, encompassing models like XGBoost and LightGBM, excels in capturing complex, nonlinear relationships and has demonstrated superior performance in genomic data analysis and survival modeling, albeit with higher computational cost^43^.

The decision boundaries established by these algorithms play a pivotal role in optimizing model performance. LR defines linear boundaries based on probability thresholds, KNN relies on proximity-based classification, SVM constructs hyperplanes that maximize class separation margins, and GB iteratively refines boundaries through ensemble learning. These boundaries are instrumental in determining the optimal combination of training parameters (e.g., regularization strength, neighborhood size, kernel functions, and learning rate), which directly influence model generalization and predictive accuracy^44^. In biomedical applications, tuning such parameters is essential for enhancing diagnostic precision, minimizing false positives, and ensuring clinical relevance. Thus, the strategic deployment of LR, KNN, SVM, and GB—each with distinct computational profiles and strengths—supports the development of robust, interpretable, and clinically actionable models, reinforcing their status as foundational tools in modern biomedical ML. All four models were also implemented in this investigation to examine the eNose sensor response of blood, urine, and stool data files for CRC patients and control participants using Python platform version 3.8.3 (Spider 4.14), provided in Anaconda Navigator 1.9.12, running on a core-i7 PC under Windows 11 (Fig. 2). The process involved training, evaluating, and testing the four classification models, calculating their classification accuracy, and reporting their respective confusion matrices. Original eNose datasets were split into 60% for training, 20% for testing, and the remaining 20% for their cross-validation, where classification accuracy on the test set was the main measure of generalization capability for the selected feature subsets. The optimal feature subset for each model was defined as the configuration that consistently achieved the highest accuracy with the fewest features across multiple runs, balancing predictive power and feature parsimony for robust model evaluation^45^.

### Data preprocessing and augmentation

In supervised learning, ML algorithms can improve their accuracy by comparing their generated output to provided correct outputs within a training dataset, often known as ‘learning from examples,’ which is primarily used for classification and regression tasks^46^. However, medical datasets are often small due to data limitations, therefore one common solution is to synthetically increase their sizes using transformations that preserve the original labels^47^. According to our recent findings^39^, Gaussian noise was found to enhance the performance of ML models; therefore, Eq. (1) was used to randomly increase the number of samples in eNose datasets^48^:

1 $$\:PG\left(z\right)=\frac{1}{\sigma\:\sqrt{2{\Pi\:}}}\:{e}^{-\frac{{\left(z-\mu\:\right)}^{2}}{2{\sigma\:}^{2}}}$$

Where z is the grey level, µ is the mean grey value, and σ is standard deviation.

A 60 × 10 × *n* high-dimensional array was generated for all eNose datasets by keeping track of the sample measurement time (60 s), the number of sensors (10), and the number of samples (n). The original 50 limited datasets for each bodily fluid as blood, urine, and stool samples were augmented to 1000 by adjusting the means and standard deviations of each dataset from 0.01 to 0.09, with an increase of 0.01 for each step.

### Statistical analysis

Quantitative analysis of CRC patients and control participants demographic, lab, and clinical characteristics was conducted using SPSS (Version 16; Chicago, IL, USA). An unpaired student *t*-test was used to compare normally distributed variables in the two groups. The results were presented as the mean plus or minus the standard deviation (± SD), and a *p*-value of less than 0.05 showed statistical significance.

PCA and LDA techniques were applied independently to eNose datasets obtained from blood, urine, and stool samples to explore latent data structures and assess class separability. The analysis yielded metrics including the top two principal variances, cumulative explained variance, and discrimination power, as a measure of classification accuracy. PCA cluster plots and correlation matrices were subsequently employed to manually derive binary classification metrics—true positives (TP), true negatives (TN), false positives (FP), and false negatives (FN)—which are critical for benchmarking unsupervised data structure separation against supervised ML model performance^32,35^.

Moreover, KNN, SVM, LR, and GB models were evaluated on both original and Gaussian augmented eNose datasets, assessing their performance through accuracy (in percentages with 95% CI), sensitivity, specificity, positive predictive value (PPV), and negative predictive value (NPV). As illustrated in Fig. 2, models’ performance was further elucidated through receiver operating characteristic (ROC) curve analysis and confusion matrix analysis, which provided detailed counts of TP, TN, FP, FN, and the area under the curve (AUC)^30,32,35^. The models’ efficacy was assessed by calculating precision (TP / TP + FP), recall (TP / TP + FN), and their harmonic mean (F1-score), which together quantify the ability to correctly identify CRC cases, minimize false positives, and balance both aspects of classification performance^39^.

## Results and discussion

CRC remains a significant global health challenge due to its asymptomatic progression and high mortality^50^. Multiple malignancies are known to induce systemic metabolic alterations, resulting in aberrant VOC profiles detectable in exhaled breath, blood, sputum, and urine^27,28,33–36,39^. This study presents a novel multi-matrix approach for non-invasive CRC detection using eNose technology, uniquely integrating blood, urine, and stool VOC profiles into a unified diagnostic framework. PCA was employed as an unsupervised dimensionality reduction technique to reveal latent data structures and facilitate group differentiation. These insights informed the application of supervised ML models, LR, KNN, SVM, and GB, which demonstrated high classification accuracy across all biological matrices. Gaussian augmentation of the VOC datasets further improved model generalizability and pattern recognition, reinforcing the eNose system’s potential as an accessabel, non-invasive screening tool for CRC. This section provides an in-depth analysis of the study’s novel findings.

### Clinical, hematological, and diagnostic findings

CRC patients in this study had a mean age of 45.61 ± 8.70 years, closely matching controls (45.22 ± 7.54 years), with balanced sex distribution, thereby eliminating age-related confounding and establishing baseline comparability. Table 2 highlights significant differences in weight and BMI (*p* < 0.01), likely reflecting disease-related cachexia or pre-diagnostic weight loss^51^. Hematologically, CRC patients exhibited numerically higher RBC counts but lower hemoglobin levels, consistent with anemia of chronic disease or occult bleeding, though reticulocyte data would be needed to confirm clinical relevance^52^. Significantly lower WBC (*p* < 0.01) and platelet counts (*p* < 0.01) suggest immunosuppression, marrow involvement, or thrombopoietic dysregulation, while hypoalbuminemia (*p* < 0.01) may indicate systemic inflammation or nutritional deficiency^52,53^. Symptomatically, patients reported high gastrointestinal burden—abdominal pain (80%), constipation (52%), diarrhea (48%), mucorrhea (40%), and rectal bleeding (60%)—alongside systemic features such as weight loss (80%) and anemia (72%). Anal pain (40%) and back pain (8%) may reflect local invasion or referred pain. Hypertension was rare (8%), while 20% of patients had a familial CRC history, reinforcing genetic predisposition^54–56^. All CRC cases were stage III (60% IIIA, 40% IIIB), consistent with delayed detection trends in Upper Egypt^57^. Early detection remains critical for curability, with removal of precancerous adenomas offering preventive benefit^58^. Current guidelines recommend initiating screening at age 45 for average-risk individuals^59–61^, using fecal occult blood tests (FIT), stool DNA-FIT, CT colonography, and colonoscopy. Despite its diagnostic superiority, colonoscopy faces barriers including cost, invasiveness, and low adherence. Emerging technologies such as eNose, which detects VOCs in biological fluids, offer a non-invasive, cost-effective alternative^28,39^. By improving compliance and enabling earlier diagnosis, eNose-based screening may help overcome current limitations in CRC detection strategies.

**Table 2:** Demographic, lab. investigations, clinical manifestations, and staging of colorectal cancer (CRC) patients as compared to control participants.

	Control participants	CRC patients
Number	50	50
Sex (M/F)	26/24	28/22
Age (y)	45.22 ± 7.54	45.61 ± 8.70
Weight (kg)	81.24 ± 12.61	76.70 ± 11.00*
Height (m)	1.69 ± 0.09	1.67 ± 0.09
BMI (kg/m^2^)	28.44 ± 4.50	27.44 ± 4.53
Hemoglobin (g/dL)	11.14 ± 2.21	10.58 ± 2.47
Red blood cells × 10^9^/L	3.41 ± 0.24	3.61 ± 0.93
White blood cells × 10^6^/L	7.14 ± 1.36	5.41 ± 2.14*
Platelets × 10^9^/L	325.27 ± 25.43	276.04 ± 103.42*
Albumin (IU/ml)	4.51 ± 0.54	4.04 ± 0.21*
Clinical manifestation *n* (%)		
Abdominal pain	—	40 (80%)
Constipation	—	26 (52%)
Diarrhea	—	24 (48%)
Mucorrhea	—	20 (40%)
Bleeding per rectum	—	30 (60%)
Weight loss	—	40 (80%)
Anemia	—	36 (72%)
Anal pain sometimes	—	20 (40%)
Back pain	—	4 (8%)
High blood pressure	—	4 (8%)
Family history	—	10 (20%)
CRC stage IIIA (TNM)	—	30 (60%)
CRC stage IIIB (TNM)	—	20 (40%)

*P < 0.01 as compared to Controls.

TNM: Tumor/Node/Metastasis; BMI: Body Mass Index.

### eNose sensor response

**Fig. 3:**
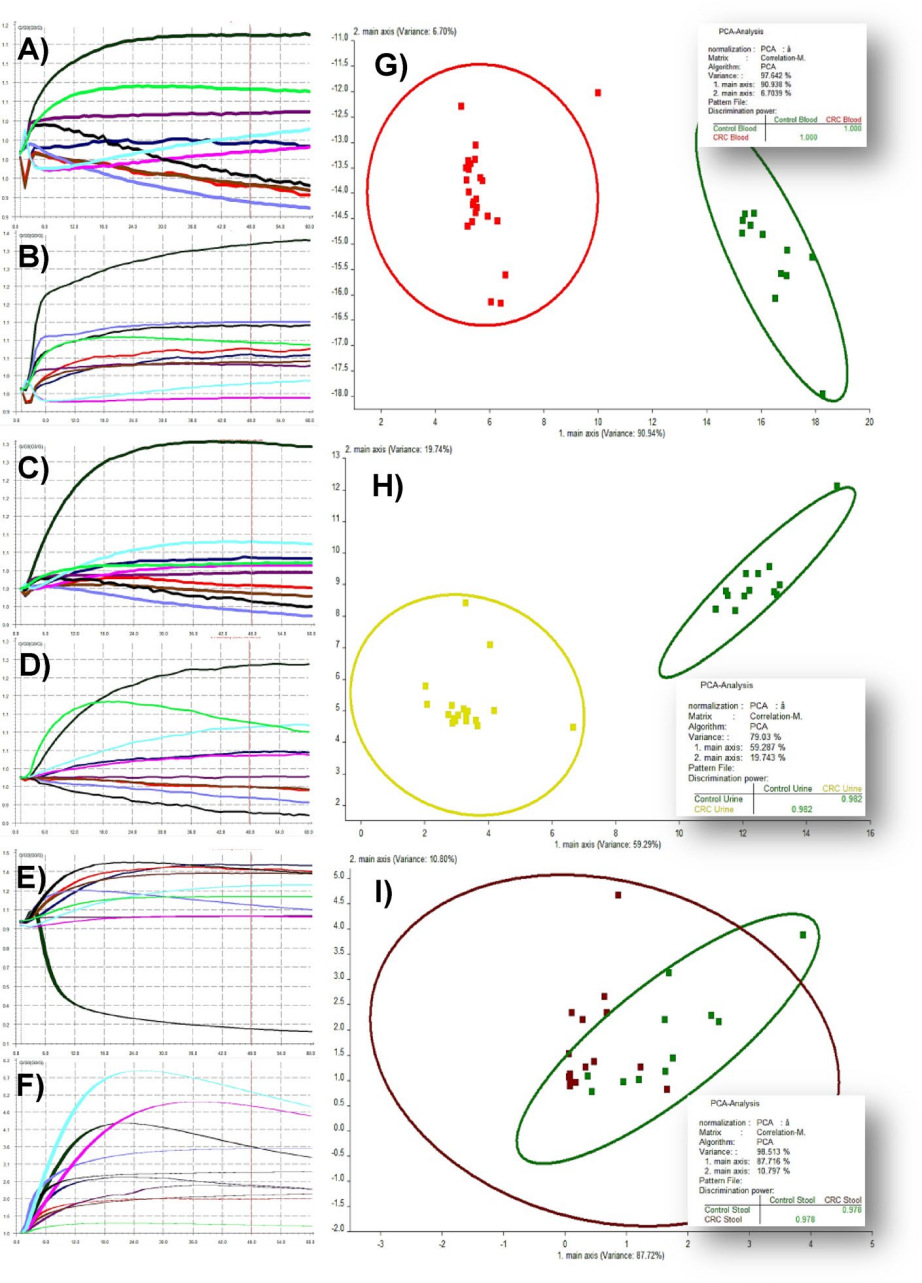
Typical eNose sensor responses in the headspace of blood, urine, and stool samples from colorectal cancer (CRC) patients (**A**, **C**, **E**) and control participants (**B**, **D**, **F**), together with principal component analysis (PCA) cluster plots, of principal component #1 on the *x*-axis against principal component #2 on the *y*-axis, for blood (**G**), urine (**H**), and stool (**I**) samples.

The typical eNose sensor responses to VOCs in the headspace of blood, urine, and stool samples from CRC patients and control participants are illustrated in Fig. 3A–F. Among the sensors listed in Table 1, S2, S4, and S10—responsive to nitrogen, hydrogen, and methane—showed elevated signals in CRC blood samples. In urine, sensors S1, S2, S9, and S10 , which detect aromatic, nitrogenous, sulfur, ammonia, and methane-related compounds, exhibited higher responses in CRC patients. Stool samples revealed increased activity in sensors S1, S3, S5, S8, S9, and S10, sensitive to aromatic compounds, ammonia, alkanes, methane, and alcohols. Together, these findings demonstrate consistent VOC elevation across all matrices in CRC patients, particularly in sensors tuned to nitrogenous, aromatic, and methane-related compounds. This aligns with prior evidence that disease-specific metabolic alterations manifest in circulating VOCs. Kim et al.^62^, using solid-phase microextraction and comprehensive two-dimensional gas chromatography-mass spectrometry, identified elevated oxetane, 2,2-dimethyl-, cyclopentane, 1-methyl-2-(2-propenyl)-, and trans-isomers in CRC blood, with high odds ratios. Additional compounds such as octanal, heptanal, 2-pentanone, and hexanal have also been reported in CRC patients^20^. Notably, 1,3-dimethylbenzene, a xylene isomer, has been detected in breath and urine and linked to CRC^20,22^. Śmiełowska et al.^63^ recently identified a CRC-associated VOC panel in breath and stool, including n-hexane, acetone, dimethyl trisulfide, and skatole, alongside elevated alcohols, nitrogenous compounds, and terpenes. These VOCs are thought to arise from colonic fermentation processes involving colonocytes, fecal microbiota, mucosal integrity, and pathogenic interactions^64,65^. Resulting VOC pattern variations are increasingly interpreted as reflections of gastrointestinal microenvironmental shifts, implicating gut microbiota dysbiosis in CRC pathophysiology^66^.

### Unsupervised model performance

Although PCA is not a classifier, its application to raw eNose datasets enabled dimensionality reduction that revealed distinct clustering patterns by suppressing noise and preserving discriminatory variance in biosignal-rich matrices^67^. The discriminative performance of PCA algorithm in separating CRC patients from controls using VOC profiles across blood, urine, and stool matrices is illustrated in Fig. 3; Table 3. PCA cluster plots (Fig. 3G–I) of principal components 1 and 2 revealed clear group separation in blood (97.64%) and stool (98.51%) samples, with comparatively lower separability in urine (79.03%). This difference may reflect the lower volatility or complexity of urine-derived VOCs, which are less separable in PCA cluster plots (Fig. 3H), consistent with findings by Safari et al.^49^. Other studies have emphasized the reliability of blood and stool VOCs, reflecting systemic biochemical alterations and localized microbial or tumor-associated signatures^68,69^, over urine, which, despite being non-invasive, suffers from dilution and renal filtration effects, explaining its lower classification performance^53,64,70^.

**Table 3:** Classification accuracy (in percentages with 95% CI) for original individual and overall eNose blood, urine, and stool datasets of colorectal cancer (CRC) patients and control participants using principal component analysis (PCA) as compared to logistic regression (LR), k-nearest neighbor (KNN), support vector machine (SVM), and gradient boosting (GB) models.

Algorithm	Blood	Urine	Stool	All datasets
PCA	97.64 (93.66–100.34)	79.03 (71.02–86.98)	98.51 (93.98–100.55)	97.95 (95.99–99.91)
LR	56.00 (42.31–68.84)	76.00 (62.59–85.70)	88.00 (76.20–94.38)	83.33 (66.44–92.66)
KNN	84.00 (71.49–91.66)	66.00 (52.15–77.56)	70.00 (56.25–80.90)	80.00 (62.69–90.49)
SVM	62.00 (48.15–74.14)	78.00 (64.76–87.25)	96.00 (86.54–98.90)	80.00 (62.69–90.49)
GB	90.00 (78.64–95.65)	86.00 (73.81–93.05)	100.00 (92.87–100.00)	90.00 (74.38–96.54)

**Fig. 4:**
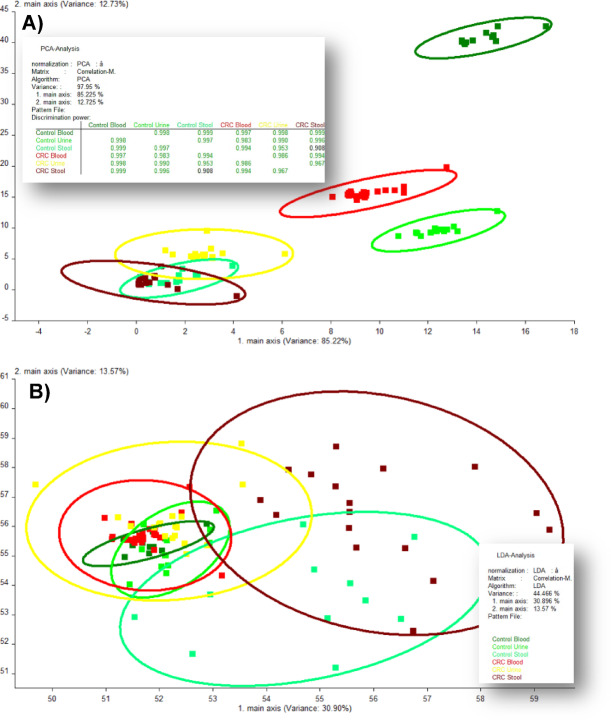
Principal component analysis (PCA) (**A**) and linear discriminant analysis (LDA) (**B**) for overall blood, urine, and stool samples from colorectal cancer (CRC) patients and control participants.

Figure 4 highlights PCA’s superior dimensionality reduction and pattern discrimination capabilities, with comparative cluster plots showing a stark contrast in overall accuracy, 97.95% for PCA versus 44.47% for LDA, and correlation coefficient values approaching 1 in Fig. 4A, validating PCA’s effectiveness in capturing CRC-specific VOC signatures. When combined with supervised methods like LDA, PCA enhanced signal discrimination in gastrointestinal cancer diagnostics, achieving up to 97% accuracy in sweat VOC-based CRC detection^64,71^. Further independent analysis in Table 4 showed that PCA yielded robust classification accuracy across all matrices, with an AUC of 0.98 and nearly 98% sensitivity, specificity, and overall accuracy, consistent with its established efficacy in VOC-based cancer diagnostics^67,72^. While its balanced PPV, NPV, and F1 score (~ 98%) confirm its effectiveness in analyzing VOC signal fluctuations and reducing high-dimensional eNose datasets. Supporting evidence showed that VOCs in blood and stool reflect systemic and localized metabolic alterations in CRC, with PCA-enhanced models achieving AUC values exceeding 0.90^63,68^. A meta-analysis of 32 studies reported pooled AUC values between 0.90 and 0.93 for VOC-based CRC detection, with PCA and ensemble models outperforming traditional classifiers^73^. These findings reinforce PCA’s translational relevance as a preprocessing tool for high-dimensional VOC datasets and support its integration into non-invasive CRC screening strategies, particularly using blood and stool matrices with consistently high signal-to-noise ratios.

**Table 4:** Metrics evaluating overall original eNose blood, urine, and stool datasets of colorectal cancer (CRC) patients and control participants using principal component analysis (PCA) as compared to logistic regression (LR), k-nearest neighbor (KNN), support vector machine (SVM), and gradient boosting (GB) models.

Algorithm	Accuracy (%) (95% CI)	Sensitivity (%) (95% CI)	Specificity (%) (95% CI)	PPV (%) (95% CI)	NPV (%) (95% CI)	F1 Score (%) (95% CI)	AUC
PCA	97.95 (95.99–99.91)	97.90 (95.09–100.71)	98.00 (95.26–100.74)	98.00 (95.25–100.74)	97.90 (95.09–100.71)	97.95 (95.99–99.91)	0.98
LR	83.33 (66.44–92.66)	93.75 (71.67–98.89)	71.43 (45.35–88.28)	78.95 (56.67–91.49)	90.91 (62.26–98.38)	85.71 (73.19–98.24)	0.83
KNN	80.00 (62.69–90.49)	68.75 (44.40–85.84)	9286 (68.53–98.73)	91.67 (64.61–98.51)	72.22 (49.13–87.50)	78.57 (63.89–93.25)	0.80
SVM	80.00 (62.69–90.49)	93.75 (71.67–98.89)	64.29 (38.76–83.66)	75.00 (53.13–88.81)	90.00 (59.58–98.21)	83.33 (70.00–96.67)	0.80
GB	90.00 (74.38–96.54)	93.75 (71.67–98.89)	85.71 (60.06–95.99)	88.24 (65.66–96.71)	92.31 (66.69–98.63)	90.91 (80.62–100.00)	0.90

CI: Confidence Interval; PPV: Positive Predictive Value; NPV: Negative Predictive Value; AUC: Area Under Curve.

### Supervised model performance using original eNose datasets

ML has become a cornerstone in oncology, enabling accurate prediction of cancer susceptibility, recurrence, and survival by integrating multi-dimensional datasets spanning genomics, imaging, clinical records, and epidemiological variables^74^. Supervised ML algorithms such as ANNs, SVM, and GB ensemble-based methods have demonstrated 15–20% improvements in predictive accuracy over traditional statistical models, facilitating robust feature selection and patient stratification essential for precision medicine^75,76^. Nonetheless, clinical translation remains challenged by limited sample sizes, high dimensionality, and inconsistent validation protocols—prompting recent emphasis on multi-modal data integration and rigorous cross-validation to enhance generalizability.

**Fig. 5:**
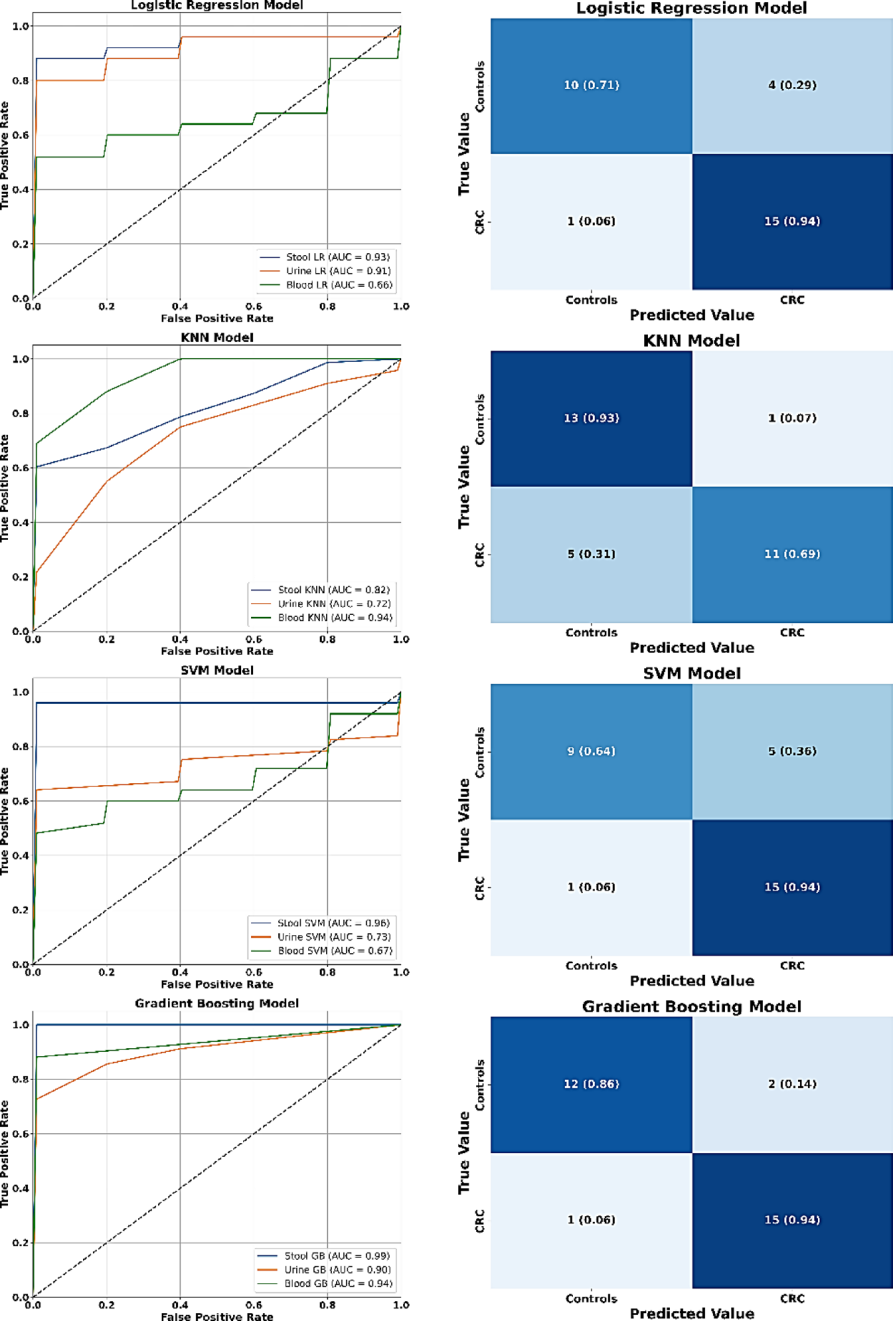
Receiver operating characteristic (ROC) curves and confusion matrix of machine learning models: logistic regression (LR) (**A**, **B**), k-nearest neighbor (KNN) (**C**, **D**), support vector machine (SVM) (**E**, **F**), and gradient boosting (GB) (**G**, **H**) for training fold (*n* = 30, 60%) of original eNose blood, urine, and stool datasets used for training from colorectal cancer (CRC) patients and control participants.

Tables 3 and 4, along with Fig. 5, provide a comparative evaluation of LR, KNN, SVM, and GB models against PCA across original eNose datasets from blood, urine, and stool samples of CRC patients and controls. LR showed limited classification performance, particularly in blood (56%), due to its linear assumptions, although it maintained strong sensitivity and NPV, making it reliable for identifying true CRC cases but prone to misclassifying controls. This reflects LR’s known limitations in modeling nonlinear VOC relationships^77^ and its specificity trade-off in complex biosensor data^67^. KNN showed moderate accuracy, excelling in specificity and PPV due to its local pattern recognition, but its reduced sensitivity and F1 score emphasize vulnerability to noise and feature scaling, especially in heterogeneous VOC datasets^78^. Despite prior success in optimized cancer models^67^, KNN’s performance here suggests potential overfitting and limited generalizability under small sample conditions. SVM yielded variable results, performing well in stool data (96%) where boundary margins were distinct, but underperformed in blood (62%) due to kernel limitations and class overlap. Its sensitivity was comparable to LR, though specificity and PPV remained suboptimal. These findings highlight SVM’s strength in high-dimensional VOC profiles and its susceptibility to overfitting in less discriminative matrices^79^. GB emerged as the most robust supervised model, consistently achieving the highest classification accuracy across all sample types, particularly in stool (100%), where its ensemble learning and iterative refinement captured subtle VOC signatures with exceptional precision. This aligns with recent evidence supporting ensemble-based models as superior classifiers in multi-modal CRC detection frameworks^73,76^.

The relatively lower performance across all models in urine datasets likely reflects weaker CRC-specific VOC signals or greater inter-individual variability, which complicate pattern recognition and model generalization. LR’s underperformance in VOC-based classification due to its linear constraints^77^ and KNN’s sensitivity to sample imbalance^78^ underscore the need for advanced preprocessing and targeted feature selection. Incorporating data augmentation strategies, such as synthetic sample generation, Gaussian noise injection, or matrix-specific oversampling, can bolster signal fidelity and enhance classification robustness, particularly in urine-based VOC analysis where signal-to-noise ratios are less favorable^49,63^.

### Supervised model performance using Gaussian-augmented eNose datasets

**Fig. 6:**
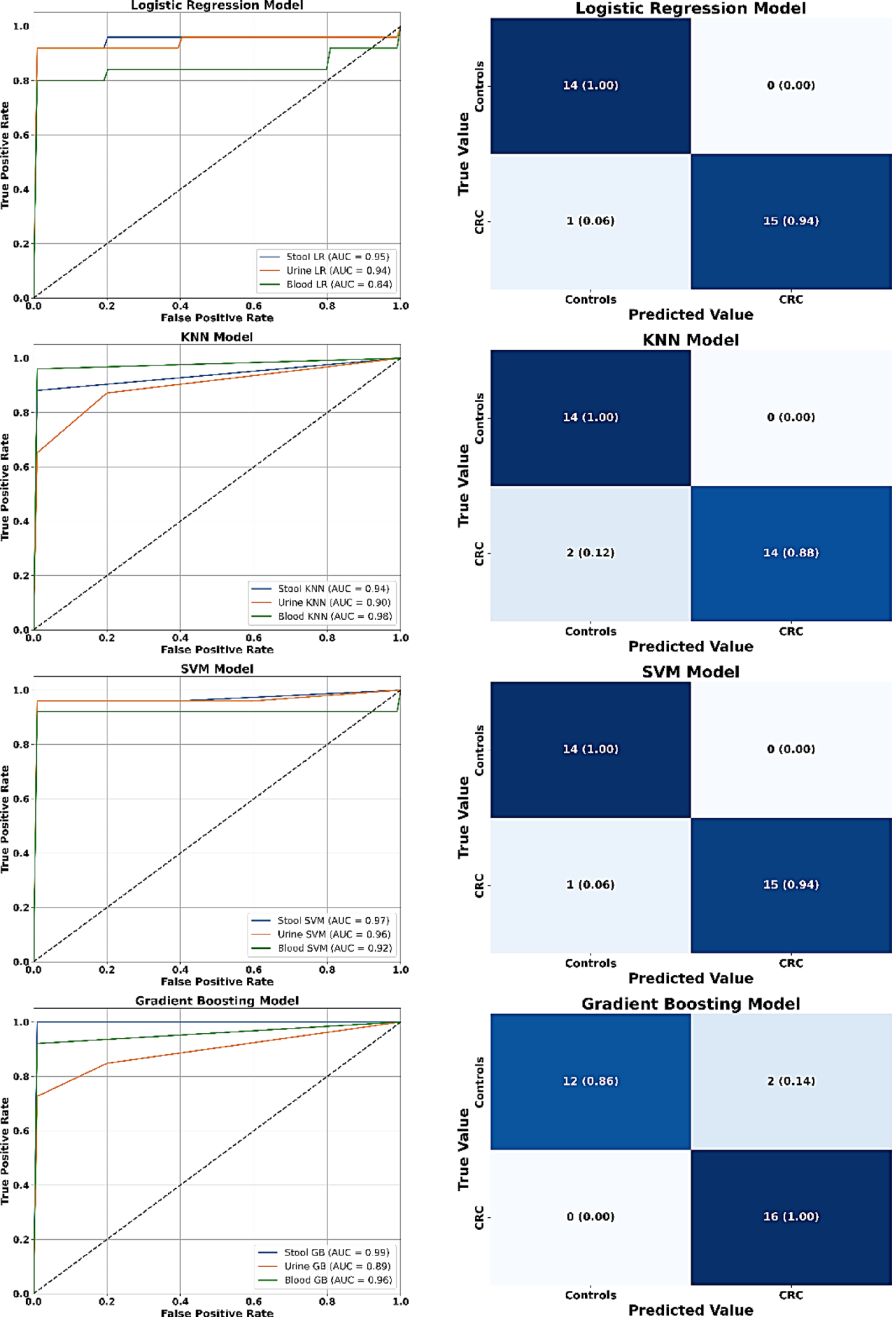
Receiver operating characteristic (ROC) curves and confusion matrix of machine learning models: logistic regression (LR) (**A**, **B**), k-nearest neighbor (KNN) (**C**, **D**), support vector machine (SVM) (**E**, **F**), and gradient boosting (GB) (**G**, **H**) for training fold (*n* = 30, 60%) of Gaussian-augmented eNose blood, urine, and stool datasets from colorectal cancer (CRC) patients and control participants.

Data augmentation is a well-established technique for enhancing ML model performance and robustness without increasing computational burden, achieved by introducing controlled variability into training datasets to improve generalization and reduce sensitivity to input noise and inconsistencies^80^. In this study, Gaussian augmentation applied to eNose-derived VOC datasets (Table 5, Fig. 6) markedly improved classification metrics across LR, KNN, SVM, and GB models, transforming previously moderate classifiers into highly reliable tools for CRC detection. LR, which initially struggled with nonlinear VOC distributions, reached 96.67% accuracy and perfect specificity and PPV post-augmentation, indicating that synthetic Gaussian noise effectively compensated for its linear modeling constraints by enhancing feature separability^77^. KNN, traditionally sensitive to data sparsity and feature scaling^78^ showed substantial gains in specificity and PPV, though its slightly reduced sensitivity (87.50%) suggests residual difficulty in capturing subtler CRC signals. SVM matched LR across all metrics, benefiting from augmented data that improved margin-based separation and reduced class overlap, consistent with its kernel-based capacity to model nonlinear VOC patterns^67,79^. GB maintained perfect sensitivity and high overall accuracy, despite its specificity slight drop to 85.71%, possibly due to overfitting to augmented CRC features, a known risk when synthetic variance inflates TP rates^67^. Thus, Gaussian augmentation enriched the VOC feature space, improving class separability and reducing model bias across biological matrices, particularly blood and stool, which offer higher VOC complexity and diagnostic yield^81^. These findings support the strategic integration of synthetic data generation in VOC-based diagnostic pipelines, especially under constraints of limited sample size or class imbalance, reinforcing the translational potential of ML-enhanced eNose systems for non-invasive CRC screening.

**Table 5:** Metrics evaluating overall Gaussian-augmented eNose blood, urine, and stool datasets of colorectal cancer (CRC) patients and control participants using principal component analysis (PCA) as compared to logistic regression (LR), k-nearest neighbor (KNN), support vector machine (SVM), and gradient boosting (GB) models.

Algorithm	Accuracy (%) (95% CI)	Sensitivity (%) (95% CI)	Specificity (%) (95% CI)	PPV (%) (95% CI)	NPV (%) (95% CI)	F1 Score (%) (95% CI)	AUC
LR	96.67 (83.33–99.41)	93.75 (71.67–98.89)	100.00 (78.47–100.00)	100.00 (79.61–100.00)	93.33 (70.18–98.81)	96.77 (90.45–100.00)	0.97
KNN	93.33 (78.68–98.15)	87.50 (63.98–96.50)	100.00 (78.47–100.00)	100.00 (78.47–100.00)	87.50 (63.98–96.50)	93.33 (84.41–100.00)	0.93
SVM	96.67 (83.33–99.41)	93.75 (71.67–98.89)	100.00 (78.47–100.00)	100.00 (79.61–100.00)	93.33 (70.18–98.81)	96.77 (90.45–100.00)	0.97
GB	93.33 (78.68–98.15)	100.00 (80.64–100.00)	85.71 (60.06–95.99)	88.89 (67.20–96.90)	100.00 (75.75–100.00)	94.12 (85.70–100.00)	0.93

CI: Confidence Interval; PPV: Positive Predictive Value; NPV: Negative Predictive Value; AUC: Area Under Curve.

### Model performance across standard and group k-fold cross-validation

Model cross-validation results (Table 6) emphasized the critical role of subject-aware validation and the efficacy of Gaussian augmentation in stabilizing VOC-based ML classifiers for CRC detection. In the original eNose datasets, performance varied widely across models and validation schemes, accuracy ranged from 64.17% (SVM, group K-Fold) to 85.83% (GB, standard K-Fold), with pronounced instability reflected in the large standard deviations, particularly for LR and SVM under grouped validation. This variability revealed susceptibility to participant-level data leakage and overestimation of accuracy when non-independent replicates were split across folds^46,74^. Grouped cross-validation consistently yielded lower performance for most models, confirming its methodological necessity for evaluating generalizability in clinical datasets with inter-individual variability^38,79^. Following Gaussian augmentation, confined exclusively to training folds, all models showed near-perfect or perfect classification accuracy across both validation strategies, with LR and SVM reaching 96.39% and both KNN and GB achieving 100.00% accuracy. The substantial reduction in standard deviation (e.g., LR: ±9.65 to ± 0.34) reflects improved robustness and boundary delineation, consistent with augmentation strategies shown to enhance hyperspectral and VOC-based classification^80^. Importantly, models previously limited by sparse or noisy VOC datasets, such as KNN and GB, exhibited flawless performance post-augmentation, indicating that synthetic feature expansion effectively mitigated prior constraints. This convergence of results across both standard and grouped cross-validation schemes reinforces Gaussian augmentation as a powerful tool for clinical deployment, where sample heterogeneity and limited cohort sizes often impair model reliability^4,20,82^.

**Table 6:** Average accuracy of standard and group k-fold cross-validation evaluating overall original eNose datasets versus Gaussian-augmented datasets using logistic regression (LR), k-nearest neighbor (KNN), support vector machine (SVM), and gradient boosting (GB) models.

Algorithm	Original eNose Data		Gaussian-augmented eNose data	
	Standard K-Fold	Group K-Fold	Standard K-Fold	Group K-Fold
LR	75.83 ± 9.65	70.00 ± 16.12	96.39 ± 0.34	96.39 ± 0.70
KNN	65.83 ± 10.67	71.67 ± 6.12	100.00 ± 0.00	100.00 ± 0.00
SVM	75.00 ± 9.86	64.17 ± 16.16	96.39 ± 0.34	96.39 ± 0.70
GB	85.83 ± 5.65	84.17 ± 4.86	100.00 ± 0.00	100.00 ± 0.00

Values are presented as the mean ± standard deviation.

### eNose sensor significance and model interpretability

**Fig. 7:**
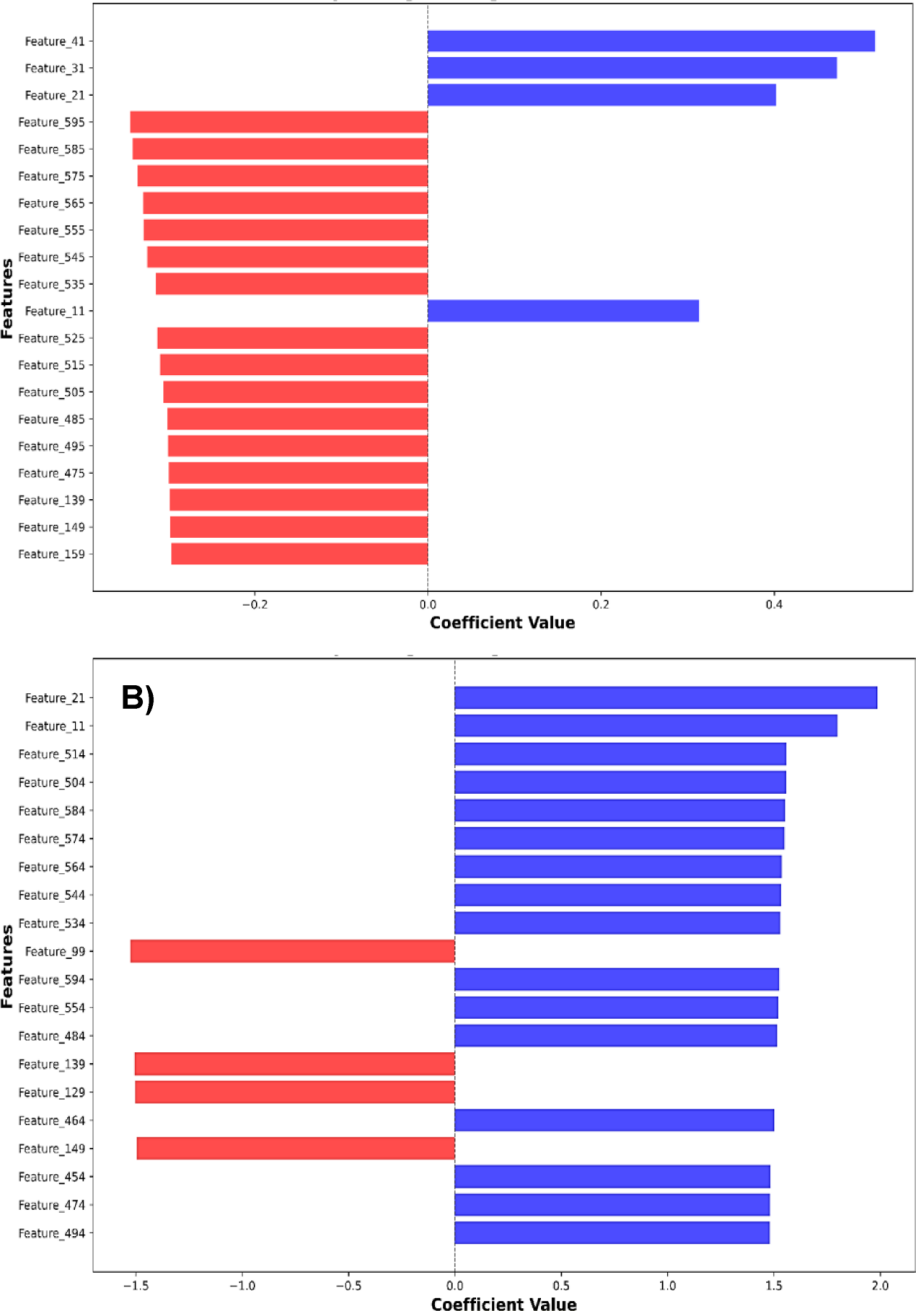
Top 20 logistic regression (LR) coefficients of features extracted from original eNose blood, urine, and stool datasets (**A**), and from Gaussian-augmented datasets (**B**) for colorectal cancer (CRC) patients and control participants.

**Fig. 8:**
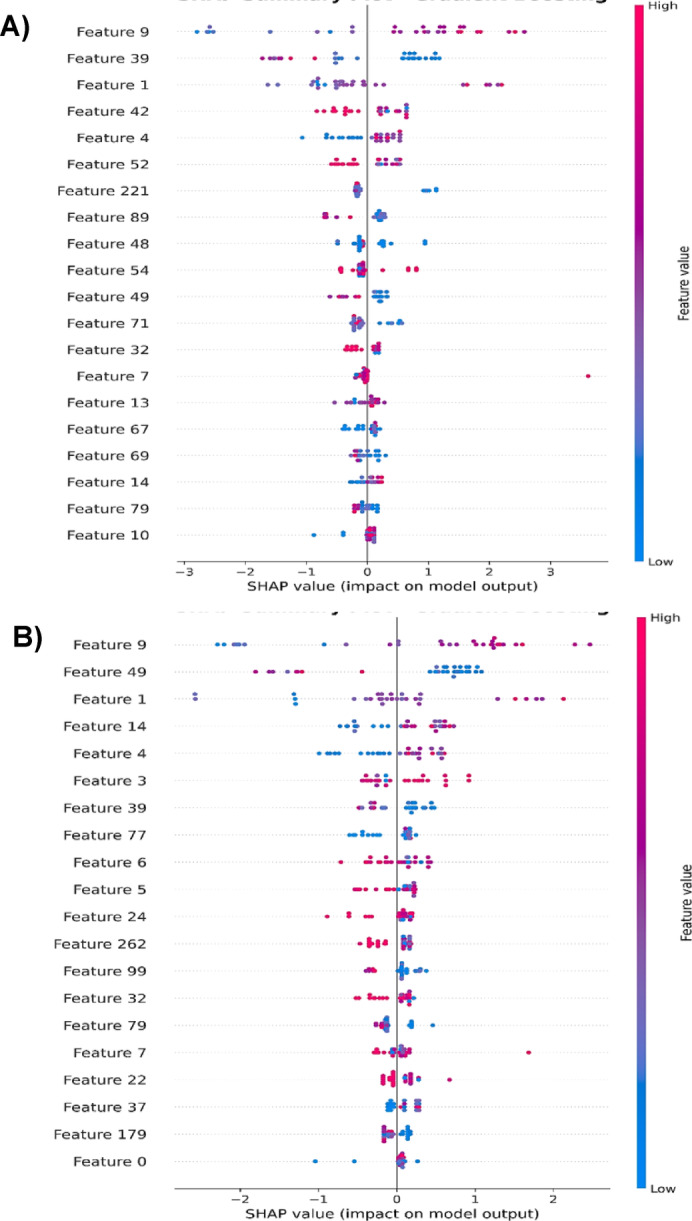
SHAP summary plot of gradient boosting (GB) shows the impact of features extracted from all original eNose blood, urine, and stool datasets (**A**), and from Gaussian-augmented datasets (**B**) for colorectal cancer (CRC) patients and control participants.

The normalized sensor importance profiles in Table 7 provide an overview of VOC-driven CRC classification across blood, urine, and stool matrices, with sensor S1 consistently dominant across all models, suggesting a robust and potentially biologically relevant VOC signal^62,64^. However, the distribution of sensor importance varies markedly by algorithm: LR and SVM exhibited balanced contributions, indicative of multivariate decision boundaries, while KNN disproportionately emphasized distal sensors (e.g., S10: 6.1282), likely reflecting sensitivity to feature scaling and noise^42,43^. GB, despite its high classification accuracy, assigned near-exclusive importance to S1, raising concerns about feature redundancy and potential overfitting, especially under Gaussian augmentation^80^. Figs. 7 and 8 further illuminate these trends. LR coefficients from the original eNose datasets (Fig. 7A) showed a balanced feature distribution, while the augmented datasets (Fig. 7B) revealed amplified weights for select sensors, suggesting that Gaussian augmentation may have exaggerated class separability or introduced synthetic bias. Similarly, SHAP plots for GB demonstrated a shift from dispersed feature contributions (Fig. 8A) to concentrated predictive influence (Fig. 8B), reinforcing the need for cautious interpretation of augmented data. These findings align with recent studies showing that SHAP-enhanced ensemble models, such as LightGB, can achieve high diagnostic accuracy while maintaining interpretability, e.g., AUCs of 0.99 (training) and 0.94 (external validation) for CRC risk prediction^83^.

**Table 7:** Normalized importance of all eNose sensor responses of all eNose blood, urine, and stool datasets of colorectal cancer (CRC) patients and control participants for logistic regression (LR), k-nearest neighbor (KNN), support vector machine (SVM), and gradient boosting (GB) models.

Algorithm	LR	KNN	SVM	GB
Sensor 1	1.0000	1.0000	0.8169	1.0000
Sensor 2	0.8425	3.0897	0.9768	0.0501
Sensor 3	0.8354	4.5385	0.9111	0.0073
Sensor 4	0.8622	5.2051	0.9351	0.0275
Sensor 5	0.8341	5.3077	1.0000	0.0103
Sensor 6	0.8141	4.8974	0.9864	0.0177
Sensor 7	0.8156	5.1795	0.9521	0.0048
Sensor 8	0.7719	5.5769	0.8637	0.0022
Sensor 9	0.7504	5.8333	0.7886	0.0050
Sensor 10	0.7758	6.1282	0.7813	0.0284

Altogether, ensemble and proximity-based models like GB and KNN outperformed linear classifiers such as LR, which, despite high sensitivity, showed lower specificity, limiting its standalone diagnostic utility^76^. PCA and SVM offered a favorable balance between interpretability and performance, consistent with prior reports on VOC-based CRC screening^67,73^. Notably, Tyagi et al.^20^ demonstrated that combining ML with eNose and GC-TOF-MS profiling enables non-invasive CRC staging using urine samples, while other studies confirm blood and stool as superior matrices due to their favorable signal-to-noise ratios^81^. Thus, these findings underscore the diagnostic promise of ML-enhanced eNose systems, particularly when paired with augmentation strategies and rigorous feature attribution analyses. Such transparency is essential for identifying candidate VOC biomarkers and ensuring clinical relevance in CRC screening applications^73,82,84^.

### Study limitations

The study’s exclusive inclusion of stage IIIA and IIIB CRC patients offered methodological consistency that strengthens VOC signal clarity and facilitated robust pattern recognition by supervised ML models. This homogeneity, however, inherently limited generalizability to early-stage disease, the most clinically relevant setting for screening, where VOC signatures are less pronounced and more variable. Since VOC signal intensities correlate with disease progression and models trained solely on late-stage datasets often overfit to robust metabolic signatures, thereby limiting their accuracy when applied to early-stage cohorts^63,68,70^. While models trained on the original eNose datasets achieved moderate performance (AUC 0.80–0.90), their diagnostic accuracy was constrained by the absence of early-stage variability.

Gaussian augmentation effectively addressed this limitation by enriching the training folds with biologically plausible VOC fluctuations, thereby enhancing model adaptability to screening contexts and improving classification metrics across all supervised ML models (Table 5). This strategy aligns with prior findings demonstrating that synthetic augmentation improves sensitivity and robustness in VOC-based cancer diagnostics^49,63^. Additionally, the slight BMI reduction observed in CRC patients (27.44 ± 4.53 vs. 28.44 ± 4.50) likely reflects the CRC-associated weight loss reported in 80% of cases. Changes in BMI are known to alter lipid metabolism, systemic inflammation, and microbiota dynamics, mechanisms which modulate VOC expression and consequently may affect classifier performance^64^. Nonetheless, the narrow BMI range across groups minimizes confounding, allowing Gaussian augmentation to focus on signal diversity rather than compensating for systemic disparities, further reinforcing its value in enhancing ML model generalizability for early-stage CRC detection.

## Conclusion

eNose technology offers a rapidly advancing, non-invasive, and cost-effective platform, particularly for colorectal and gastric cancers early detection and treatment monitoring^82^. Combining ML-enhanced sensor interpretation and denoising algorithms can improve VOC signal fidelity, while its affordability and portability make it especially valuable for early detection and point-of-care use in resource-limited settings^84^. In this study, the eNose could differentiate CRC patients from controls by analyzing distinct VOCs profiles, establishing a highly accurate method for group separation. PCA downstream analysis showed high classification accuracy of blood, urine, and stool samples, emphasizing its robust performance in the non-invasive detection of CRC. Integrating eNose system with supervised ML models—LR, KNN, SVM, and GB—efficiently detected CRC by analyzing distinct VOC profiles in all biological matrices. The successful application of different ML models was evidenced by their robust performance, particularly after Gaussian augmentation of original eNose datasets, confirming their efficacy in pattern recognition for CRC detection across all matrices. Ensemble and proximity-based models, specifically KNN and GB, demonstrated superior accuracy and generalizability compared to linear classifiers like LR. Notably, blood and stool samples yielded particularly excellent results, indicating superior diagnostic accuracy of CRC. These findings pinpoint the significant potential of eNose based on ML analysis systems to introduce a globally accessible, non-invasive, and cost-effective method for CRC detection, characterized by both high sensitivity and specificity.

## Data Availability

Metadata used and/or analyzed during the current study will be made available from the corresponding author on reasonable request.

## Declarations

**Institutional review board statement**.

This study was approved by the Ethics Committees at both the Medical Research Institute and the Faculty of Medicine at Alexandria University in Alexandria, Egypt, approved the study protocol with Approval No. 00012098. All patients had given their informed written consent stating the title, procedure, and purpose of the study. All procedures were strictly conducted in accordance with the Declaration of Helsinki. The security and privacy of all participants’ personal data were ensured by adhering strictly to privacy and ethical rules throughout all data handling procedures.
